# Label-free electrochemical cancer cell detection leveraging hemoglobin-encapsulated silver nanoclusters and Cu-MOF nanohybrids on a graphene-assisted dual-modal probe

**DOI:** 10.1038/s41598-023-49418-1

**Published:** 2023-12-11

**Authors:** Ali-Akbar Zare, Hossein Naderi-Manesh, Seyed Morteza Naghib, Mojtaba Shamsipur, Fatemeh Molaabasi

**Affiliations:** 1 Department of Nanobiotechnology, Faculty of Biological Sciences, Tarbiat Modares University, Tehran, Iran; 2 Department of Biophysics, Faculty of Biological Sciences, Tarbiat Modares University, Tehran, Iran; 3 Nanotechnology Department, School of Advanced Technologies, Iran University of Science and Technology (IUST), Tehran, Iran; 4 Department of Chemistry, Razi University, Kermanshah, Iran; 5 Biomaterials and Tissue Engineering Research Group, Department of Interdisciplinary Technologies, Breast Cancer Research Center, Motamed Cancer Institute, ACECR, Tehran, Iran

**Keywords:** Breast cancer, Biomaterials, Bioanalytical chemistry, Sensors

## Abstract

Breast cancer detection at an early stage significantly increases the chances of successful treatment and survival. This study presents an electrochemical biosensor for detecting breast cancer cells, utilizing silver nanoclusters encapsulated by hemoglobin and Cu (II)-porphyrin-metal organic framework (BioMOF) in a graphene-incorporated nanohybrid probe. This Hb-AgNCs@MOF-G probe demonstrates high electrochemical activity, superior dispersity, porosity, and a large surface area for effective functionalization. Using a green ultrasonic-assisted stirring method, we fabricate ultra-small 5 nm particles that readily immobilize on a glassy carbon electrode, generating a detection signal when interacting with ferricyanide/ferrocyanide redox probes. The resulting immunosensor detects as few as 2 cells/mL using Electrochemical Impedance Spectroscopy (EIS) “signal on” and 16 cells/mL via Square Wave Voltammetry (SWV) “signal off”, within a broad range of cell concentrations (10^2^–5 × 10^4^ cells/mL). Our designed sensor shows improved selectivity (5- to 16-fold) and robust detection in human blood with a recovery efficiency between 94.8–106% (EIS method) and 95.4–111% (SWV method). This sensor could streamline early cancer diagnosis and monitor patient treatment without requiring labelling or signal amplification. As a pioneering endeavor, we've utilized integrated porous MOFs with Hb-encapsulated silver nanoclusters in cancer detection, where these components collectively enhance the overall functionality.

## Introduction

As a disease, 25% of cancers and the cause of death of women is breast cancer (BC)^1^. According to statistics, the incidence of BC is 40.72 out of every 100,000 women in Iran^2^. In high-income societies, the death rate from BC has decreased by 40%. This means that annual BC mortality can be reduced by 2 to 4 percent per year. If only a 2.5 percent reduction in annual mortality between 2020 and 2040, 2.5 million deaths can be prevented. As a goal, the Global Breast Cancer Initiative (GBCI) has stated a reduction of 2.5%/year^3^. To realize this goal, early detection increases the chance of survival. Therefore, the improvement of a highly sensitive, rapid and cost-effective technique is essential^4^. Among the diagnostic methods, mammography cannot detect benign, malignant lesions and even up to 25% of cancers. A biopsy is also necessary to confirm the diagnosis^5^.

Detection of cell surface biomarkers as well as circulating tumor cells (CTCs) is a non-invasive way to diagnose cancer^6,7^. The transmembrane kinase receptor known as HER2 is overexpressed in 15–30% of cases^8–11^. Herceptin is a drug prescribed to patients in case of accurate measurement of HER 2 + cells^12,13^. In the case of identification of HER2 + cancer cells there are various techniques such as routine immunohistochemistry and in-situ hybridization fluorescence^12,14,15^. However, electrochemical sensors are among the most desirable devices for cancer detection development in terms of simplicity, sensitivity, and cost^16,17^. In this way, graphene sheet-based nanoplatforms are one of the interesting electrochemical sensing interface candidates because of desirable flexibility, high specific surface area, high conductivity, and unique chemical stability^18–22^. As known, oxidation of graphen to create carboxyl functional groups with aim of connection to recognition elements (e.g. antibody, aptemer) decreased its conductivity, which is not suitable for electrochemical measurements. One of effective ways that has recently received attention to produce functional graphene nanosheets by keeping conductivity is sonication of graphite powder as natural material in the presence of a protein solution resulting in high-stable graphene aqueous dispersions together with amino acid functional groups^23^.

Protein-protected noble metal nanoclusters with excellent conductivity, ultra size, core/shell structure, and simple one-step green synthesis have been successfully utilized as electrochemical sensing interfaces for sensitive detection of glucose^24^, H_2_O_2_^25^, KB cells^26^ and retinal-binding protein^27^. Metal nanoclusters composed of several metal atoms exhibit excellent optical-electrical properties and good biocompatibility compared to bigger size metal nanoparticles (MNPs) because of discrete energy levels near to the electron Fermi wavelength^28^. The excellent protecting and reducing ability of hemoglobin (Hb) as a typical multi-cofactor protein with two heme-containing dimers to synthesize ultra-small-sized nanoclusters and crystalline porous nanostructures has been previously demonstrated by our group^29^. Moreover, the high-performance electrochemical properties of Hb-stabilized metal nanoclusters have been confirmed in electrocatalytic oxygene reduction reaction (ORR)^30^ and ethanol oxidation^31^ biosensing of BCR/ABL fusion gene^32^ and H_2_O_2_^33^. In all of which, some factors play important roles for inducing electroactivity of Hb-stabilized NCs including (i) presence of metal clusters with high surface area together with Fe as an oxophilic center which can modify the electronic environment of metal clusters; (ii) 3D shape, core–shell and peculiar electronic structure of metal nanoclusters which is comparable to pure metal; (iii) the effective protein template coverage which also allows the high dispersity of clusters. However, there are few reports in relation to the applying of protein-stabilized graphene and/or protein-stabilized NCs/graphene nanohybrids, assisted by ultrasonic process, as electrosensing interface.

Metal–organic frameworks (MOFs) with universal framework functions including large specific area, biocompatibility, chemical tunability, well-defined accessible porosity, high metal bonding interactions together with various grafting amine and carboxyl groups and good adsorption characteristics which could help the co-immobilization of biological ligands and metal ions, could be considered as an another desirable electrosensing platform. However, the low conductivity and electrical reactivity, and instable structure of MOF in aqueous solution limit their electrochemical biosensing applications. To overcome these shortcomings, MOFs can be assembled with conductive materials, such as carbon nanotubes, graphene, carbon nanofibers, carbon blocks, and/or metal nanoparticles. The resulting MOF-based carbon or metal nanocomposites presents high stability, versatility, dispersibility, improved mechanical strength, richer active sites and higher surface area with highly ordered porosity arrangement. These allow more availability of active sites and increment of the electron transfer rate, so higher electrocatalytic activity and electrical conductivity. In fact, the overpotential of oxidation and reduction reactions could be decreased as a result of size and morphology of carbon nanomaterials and noble metal nanoparticles and thus the sensitivity of detection enhances^34^. In this regard, some examples of MOF-based nanocomposite can be mentioned: Ag/MIL-101 for monitoring tryptophan^35^, Cu-MOFs showing an improvement in glucose oxidation activity^36^, Cu-based MOF/graphene for electrochemical sensing of H_2_O_2_ and ascorbic acid^37^, Nucleic acid-functionalized metal–organic framework for ultrasensitive determination of carcinoembryonic antigen (CEA)^38^ and also silver nanoclusters encapsulated into ZIF-8 MOFs that has been used to detect copper in blood^39^. However, no report has been published so far which applied MOF-based nanocomposite consists of both carbon and noble metal nanomaterials for direct evidence of cancer cell detection.

Improving sensitivity, reproducibility and increasing specificity is one of the main challenges in the development of electrochemical biosensors. The development of new nanomaterials and its application as a substrate in designing new electrochemical nanobiosensors can amplify the raw signal and overcome these limitations^40^. This is also very important in relation to the measurement of tumor markers because of high matrix interference effect of the human serum. Although the use of antibodies can strengthen the sensor specificity and increase its sensitivity, the numerous substances present in the human serum sample have an obvious effect on the sensitivity and cause it to decrease. This challenge is even greater in the case of a whole blood sample for cancer cell detection, so that the signal amplification methods usually need to be adopted for the design of some biosensors. In this regard, one of the best ways is to develop nanocomposites with high-efficiency components to create high surface area, numerous functional groups and good conductivity without any modification, so that nanocomposites can act as a label free electrochemical platform to increase sensor sensitivity and accuracy^41^.

**Figure 1:**
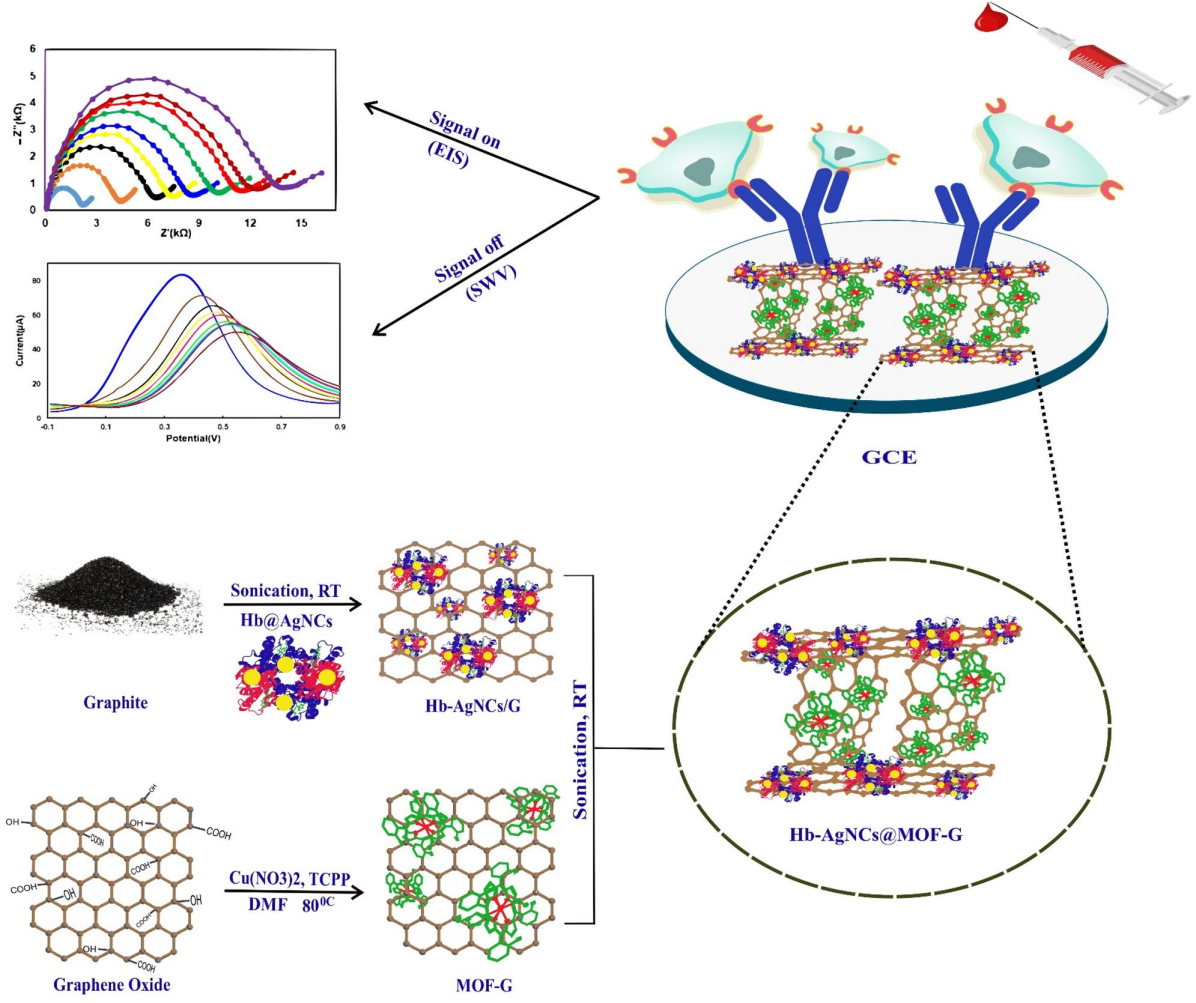
Schematic illustration of breast cancer cell detection with the designed Hb-AgNCs@MOF-G Nanohybrid .

In this study, for the first time, we report the use of novel Hb-AgNCs@MOF-G nanocomposite for the immobilization of anti-HER2 on a glassy carbon electrode (GCE) and its bioapplication for fabrication of a label free electrochemical immonusensor for the detection of HER2-positive breast cancer cells based on both Square Wave Voltammetry “signal off” and impedimetric “signal on” methods (Fig. 1). The new proposed Hb-AgNCs@MOF-G as an electrointerface sensing bioplatform presents general advantages including large specific surface area, high electrochemical activity, good biocompatibility, high dispersity and stability in aqueous solution and easy functionalization. In fact, these great advantages from simultaneous present of ultra-size silver nanoclusters stabilized by hemoglobin as an oxophilic center, highly porous BioMOF and graphene nanosheets as a strong conductive carbon material on one hand and high surface area as well as abundant functional groups in the protein on the other hand help to increase the attachment of antibody on the surface electrode, leading to an improved sensitivity and great specificity of the designed immunosensor for diagnosing cancer cells. The developed nanocomposite could be used not only for designing other immunosensor but also for enzyme/aptamer sensors, and other bioapplications such as drug delivery and medical imaging.

## Experimental section

### Materials and reagents

Phosphate-buffered saline (PBS), bovine serum albumin (BSA), 1-Ethyl-3-(3-dimethylaminopropyl)carbodiimide (EDC), NHS (N-hydroxysuccinimide) (NHS), AgNO_3_, ethanol and NaOH were purchased from Sigma-Aldrich. Potassium ferricyanide (K_3_Fe(CN)_6_) and potassium ferrocyanide (K_4_[Fe(CN)_6_]), Copper (II) nitrate trihydrate (99%) (Cu(NO_3_)_2_.3H_2_O), sulfuric acid (95%) (H_2_SO_4_), graphite powder (300 mesh, 99.99%), hydrogen peroxide (35%) (H_2_O_2_), sodium nitrate (NaNO_3_), potassium permanganate (KMnO_4_), pyrrole, propionic acid, 4-carboxybenzaldehyde, and dimethylformamide (DMF) were supplied from Merck. All the solutions were prepared in deionized water. Human monoclonal anti-HER2 antibodies (Herceptin) were purchased from AryoGen Pharmed Co. (Iran). All substances were utilized just as they were given without additional purification. The SKBR3, MCF-7, HFF, and MDA-MB231 cell lines were purchased from Pastor Institute (Iran).

### Apparatus

The Carl Zeiss device was used to capture the FESEM pictures. CM120 (Philips, Netherlands) was used for the TEM characterization at 100 kV. Measurements of EDS and elemental mapping were made (EDAX, USA) with the aid of a microanalysis system and a SEN (Hitachi E-1010, Horiba Ex-250). The absorption spectra were recorded on a model Scinco UV S-2100 (Cinco, Korea) in the wavelength range of 250 to 600 nm. The samples' hydrodynamic diameters and surface charges were determined using a Zetasizer Nano (Malvern, UK) setup with a helium–neon laser. FTIR and Raman spectra of samples were carried out on an IR spectrometer (8500S SHIMADZU) at frequencies ranging from 400 to 4000/cm and SENTERA Raman spectrometer (Bruker, Germany) with an excitation of 780 nm laser light, respectively. Porosity and surface area of the materials were recorded using Brunauer–Emmett–Teller (BET) method from N2 adsorption and desorption isotherms which were measured on a BELSORP-miniII system. Cyclic Voltammetry (CV), Electrochemical Impedance Spectroscopy (EIS), and Square Wave Voltammetry (SWV) were carried out using palmsens 2 and PSTrace (v.4) software. All electrochemical tests were conducted in 0.1 M PBS containing 5 mM K_3_ [Fe (CN)_6_]/K_4_ [Fe (CN)_6_] (1/1), while manufactured nano biosensor served as the working electrode, a platinum wire as the counter electrode, and Ag/AgCl (sat. KCl) as the reference electrode. CV curves were recorded within − 0.4 to 0.8 V vs. Ag/AgCl with a scan rate of 100 mV/s. EIS testing was completed with a potential amplitude of 10 mV and a frequency range of 0.01 Hz to 10 kHz.

### Synthesis of graphene-based hemoglobin-capped Ag nanoclusters (Hb-AgNCs/G)

In this work, Hb was selected as the stabilizing/reducing agent for the synthesis of fluorescent Ag nanoclusters (AgNCs) which could be easily prepared and extracted with high purity (> 95%) from human blood, according to the William and Tsay method^42^.

At first, the Hb-AgNCs was synthesized by the addition of an aqueous AgNO_3_ (10 mL, 0.5 mM) to a Hb solution (10 mL, 0.11 mM), followed by adding NaOH (2.0 mL, 1000 mM) after 10 min and then led to vigorous stirring for 5 days at 37 °C. Solution was subsequently centrifuged (10000 g) to remove the large silver nanoparticles^29^.

At next step, Hb-AgNCs/G was synthesized as follow: 0.1 g of graphite powder was dispersed into 10 mL of as-prepared Hb-AgNCs solution and then sonicated for 7 h. The water bubbles that form and burst under acoustic cavitation of ultrasonic waves create shock waves. This allows the graphite to become layered and broken up. After that, the Hb-AgNCs were interacted and stabilized with few-layer graphene nanosheets. In fact, the hydrophobic groups of Hb protein could be effectively adsorbed due to the hydrophobic graphene's enormous surface area, whereas the hydrophilic segments of Hb interacted with water molecules^32^. Before separating the black supernatant from the sediment, the sonicated mixture was let to stand for 24 h to allow some of the big graphene aggregates and graphite particles to settle. After that, the separated supernatant was centrifuged for 30 min at 3000 rpm to result in a stable aqueous graphene dispersion^32^.

### Preparation of graphene oxide (GO)

Graphene oxide was prepared according to Hummer’s method^43^. First, 1 g of graphite powder and sodium nitrate and 45 mL of sulfuric acid was poured into a 250 mL round bottom flask and stirred for 15 min in an ice bath. In the next step, 8 g of potassium permanganate was gradually added until its temperature reached 25 °C. The mixture was then put into a water bath at 40 °C and agitated for 60 min. After that, the temperature was reached to 95 °C and 75 mL of deionized water was gradually added while 15 mL of H_2_O_2_ 30% was used to treat the solution. The mixture was then vacuum-dried at 60 °C after being filtered and rinsed with deionized water until the pH reached 7.0.

### Synthesis of meso-tetra (4-carboxyphenyl) porphine (TCPP)

TCPP was produced using a prior study's methodology^44,45^. In a nutshell, a 250 mL round-bottomed flask was filled with 10 mmol (1.5 g) of 4-carboxybenzaldehyde, 10 mmol (newly distilled pyrrole), and 100 mL of propionic acid. The mixture was then agitated for 60 min at 140 °C and after allowed to stand at room temperature in order to separate the bigger particles and create a precipitate. The mixture was filtered and purified with ethanol. The necessary TCPP purple solid was obtained.

### Synthesis of Cu-TCPP metal–organic framework (Cu-MOF)

Solvothermal synthesis was used to create Cu-TCPP MOF. Standardly, 21.6 mg of Copper (II) nitrate was combined for 30 min with 23.7 mg of TCPP, 4.5 mL of DMF, and 1.5 mL of ethanol. The resultant suspension was placed into an autoclave with a Teflon coating and heated for 24 h at 80 °C. It then cooled to body temperature. Cu-TCPP MOF purple powder underwent numerous ethanol washes to remove impurities and then dried for 15 h at 60 °C^46^.

### Synthesis of Cu (II)-porphyrin MOF/ graphene oxide (MOF-G)

21.6 mg of Cu (NO_3_)_2_, 23.7 mg of TCPP, 4.5 mL of DMF, and 1.5 mL of ethanol were mixed to create MOF-G. The suspension was agitated for 30 min. The mixture was then added to a GO suspension (1 mg/mL), heated to 80 °C for 24 h, and then cooled to ambient temperature in an autoclave coated with Teflon. To purify the MOF-G nanocomposite, a dark purple fine powder was produced and washed numerous times with ethanol and dried for 15 h at 60 °C^47^.

### Preparation of Hb-AgNCs@MOF-G nanocomposite

The Hb-AgNCs/G solution (1.5 mg/mL) and MOF-G (1 mg/mL) were mixed followed by ultrasonically agitating for 15 min and then allowed to continue under vigorous stirring for 24 h at room temperature to form a homogeneous suspension.

### Fabrication of the electrochemical sensors

The surface of glassy carbon electrode (GCE) after cleaning with alumina powder was dropped by 10 μL of as-prepared nanocomposite and then kept at 4 °C for 24 h. After that, the modified surface of electrode was washed with dionized water and activated using an EDC/NHS linker solution (400 μM/100 μM) for 3 h at room temperature. To identify HER2 + cancer cells, this was followed by washing with PBS and functionalized with 10 μL of anti-HER2 solution (1 mg/mL) during overnight incubation at 4 °C. The resulting functionalized electrode surface as Ab/Hb-AgNCs@MOF-G/GCE sensor was rinsed with PBS to eliminate non-specific adsorption. To prevent interaction of the activated carboxyl groups that are not coated with the amino groups of Herceptin, the biosensor was incubated in 5% BSA solution for 30 min. Then, three PBS rinses was performed on the surface of electrode and stored at 4 °C until use.

#### Cell culture and detection

The normal Human foreskin fibroblasts (HFF) cell line (with an expression level of 0–1) and the BC cell lines (SKBR3, MCF-7, MDA-MB231) were grown in DMEM, 10% FBS, and 1% antibiotic (penicillin–streptomycin) at 37 °C in a humidified incubator (5% CO_2_, 95% humidity)^48^. After three days of culture, the cells were harvested with 1% trypsin/EDTA. To identify cancer cells using the developed nano biosensor, 10 μL of a cell solution with different concentrations was drop-casted and immobilized on antibody-modified surface of the electrode and stored during 20 min at room temperature. The electrode was then washed with PBS after sufficient incubation.

To confirm the potential bioapplication of the proposed design, human blood obtained from one healthy and two patients was used. For this reason, SKBR3 cells were spiked in ficoll-treated blood samples at concentrations of 10^2^,10^3^, 6 × 10^3^, 16 × 10^3^ and 66 × 10^3^ cells/mL. It should be noted that all experiments were performed in accordance with relevant guidelines (Declaration of Helsinki) and the study was approved by the Ethics Committee of Tarbiat Modares University. Informed consent was also obtained from the human participants of the study.

## Results and discussion

### Characterization of Hb-AgNCs@MOF-G nanocomposite

**Figure 2:**
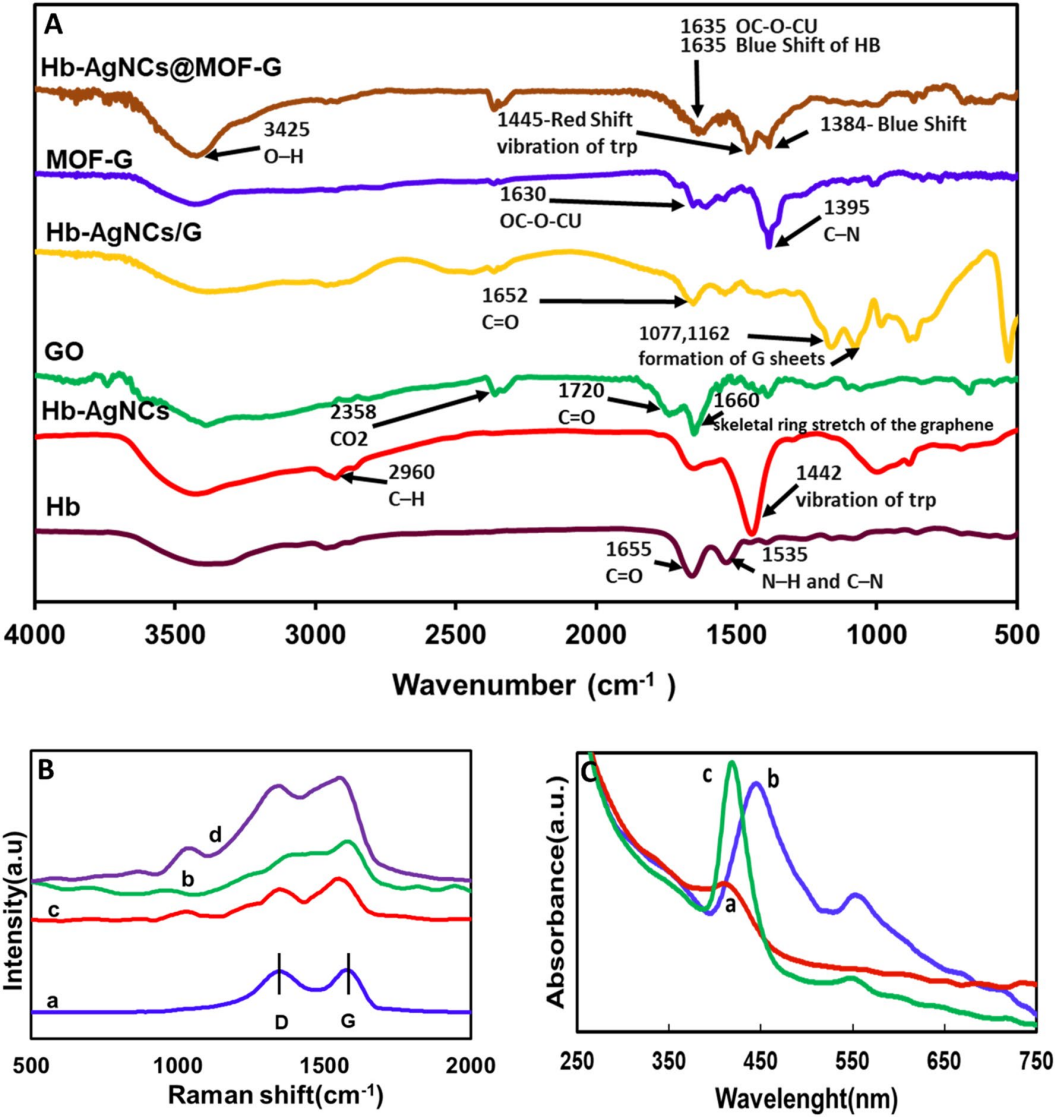
(**A**) FTIR analysis for the materials: Hb, Go, Hb-AgNCs, Hb-AgNCs/G, MOF-G, Hb-AgNCs@MOF-G; (**B**) Raman spectra data for the (**a**) GO, (**b**) Hb-AgNCs/G, (**c**) MOF-G and (**d**) Hb-AgNCs@MOF-G; (**C**) UV–Vis spectra of (**a**) Hb-AgNCs/G, (**b**) MOF-G and (**c**) Hb-AgNCs@MOF-G.

The FTIR of pure Hb, Hb-AgNCs, GO, Hb-AgNCs/G, MOF-G, and Hb-AgNCs@MOF-G were recorded to investigate the nanocomposite's formation mechanism (Fig. 2A). Since the amide bands are particularly sensitive to environmental change and distinctive H-bond patterns, the FTIR spectra were recorded to offer information on the secondary structural change of Hb before and after metal nanocluster encapsulation. The measured IR spectra indicate the following primary bands: amide I C=O bending (1600–1680 cm^-1^), amide II band (1500–1620 cm^-1^) coming from N–H, C–N bending and saturation C–H at 2960 cm^-1^. Other bands include the stretching vibration of the N–H of amide group at 3425 cm^-1^ and the aromatic amine C–N at 1395 cm^-1^. As seen, the IR of the Hb-AgNCs differed significantly from the IR spectra of the natural protein. The shape and peak position of the amide I band (1658 cm^-1^) of Hb are almost the same after the synthesis of AgNCs, although the intensity decreases. However, the amide II (1535 cm^−1^) and aromatic amine bands (1395 cm^−1^) in free Hb are disappeared in Hb-AgNCs. Furthermore, when nanoclusters are formed at high pH, the production of Hb-AgNCs increases the peak intensity centered at 1442 cm^-1^, which could be related to the vibration of tryptophan (Trp). The decrease in intensity of the amide I and stretching vibration of the N–H amide group at 3425 cm^-1^, indicates that there has been a significant change in the conformation of Hb from the free state, meaning that fewer helical structures are present as a result of interaction with the AgNCs, and the notable changes of the amide II band and the aromatic amine band are responsible for the binding of silver ions with protein via free amine groups. In the case of Hb-AgNCs/G, two peaks with centers at 1652 and 1540 cm^-1^ can be ascribed to amide I C=O stretching and amide II N–H bending and C–N stretching of Hb. Furthermore, the production of graphene sheets can be confirmed by two peaks seen at 1077 and 1162 cm^-1^. The produced nanocomposites of MOF-G exhibit a peak at 1008 cm^-1^ and two peaks at 1630 and 1400 cm^-1^ due to the establishment of coordination bonds between the carboxyl groups of the TCPP ligand and the Cu atom and also correspond to the metalation of the porphyrin ring. Moreover, the absorption band at 1660 cm^-1^ might be connected to the graphene sheets' skeletal ring stretch.

For the Hb-AgNCs@MOF-G, both absorption peaks of Hb-AgNCs-G and MOF-G are seen as follow: the 1064 and 1164 cm^-1^ from graphene sheets in Hb-AgNCs/G and MOF-G, 1384 cm^-1^ from C-N bending vibrations of aromatic amine with a blue-shifted compared to the free Hb, and 1455 cm^-1^ from Trp vibration with a red-shifted compared to the free Hb. In addition, 1635 might be ascribed to amide I C=O stretching of Hb with blue shifting compared to Hb-AgNCs/G, as well as coordination bonds among C=O groups of the TCPP ligand and Cu atoms in MOF of the nanocomposite. Furthermore, the detected peaks at 2960 and 3423 cm^-1^ are caused by the stretching vibration of the saturated C–H group, as well as stretching vibrations of the N–H amide and O–H groups in the Hb and MOF of the nanocomposite. In the nanocomposite structure, the amide II (1540 cm^−1^) found in free Hb-AgNCs/G vanished. The results show that Hb-AgNCs@MOF-G has been successfully formed and could be employed as a new electrochemical interface for cancer cell diagnosis.

Figure 2B depicts the Raman spectra of material including Hb-AgNCs/G, MOF-G, and Hb-AgNCs@MOF-G. Raman spectroscopy is an efficient approach for determining the homogeneity and authenticity of carbon composites, with the disorder (D) and graphite (G) peaks corresponding to carbon sp3 and sp2 stretching^49,50^. The peaks of graphene, which are seen at 1339 and 1591 cm^-1^, are present in all graphene-based sample spectra, as shown in Fig. 2B, demonstrating the production of graphene nanosheets^51,52^.

For further characterization, the UV–vis spectra of graphene samples were also recorded. As shown in Fig. 2C, the UV–vis spectroscopy demonstrates an absorption band around 415 nm belonging to the Soret band, which can be related to porphyrin in hemoglobin. Also, in the MOF-G sample, there is a Soret band in the region of 440 nm and three Q bands in 504, 530, and 556 nm. In the developed nanocomposite sample, the Soret band related to porphyrin in the region of 420 nm is still present with a slight increase in intensity. In addition, the Soret band was blue-shifted compared to MOF-G, indicating changing the environment of porphyrin group after nanohybrid formation. In addition, the intensity and number of Q bands reduced as results of interaction of MOF-G with Hb/AgNCs-G. The structural changes of the materials after mixing and ultrasound frequency on them to produce the final electrochemical nanocomposite demonstrates the successful formation of nanohybrid.

**Figure 3:**
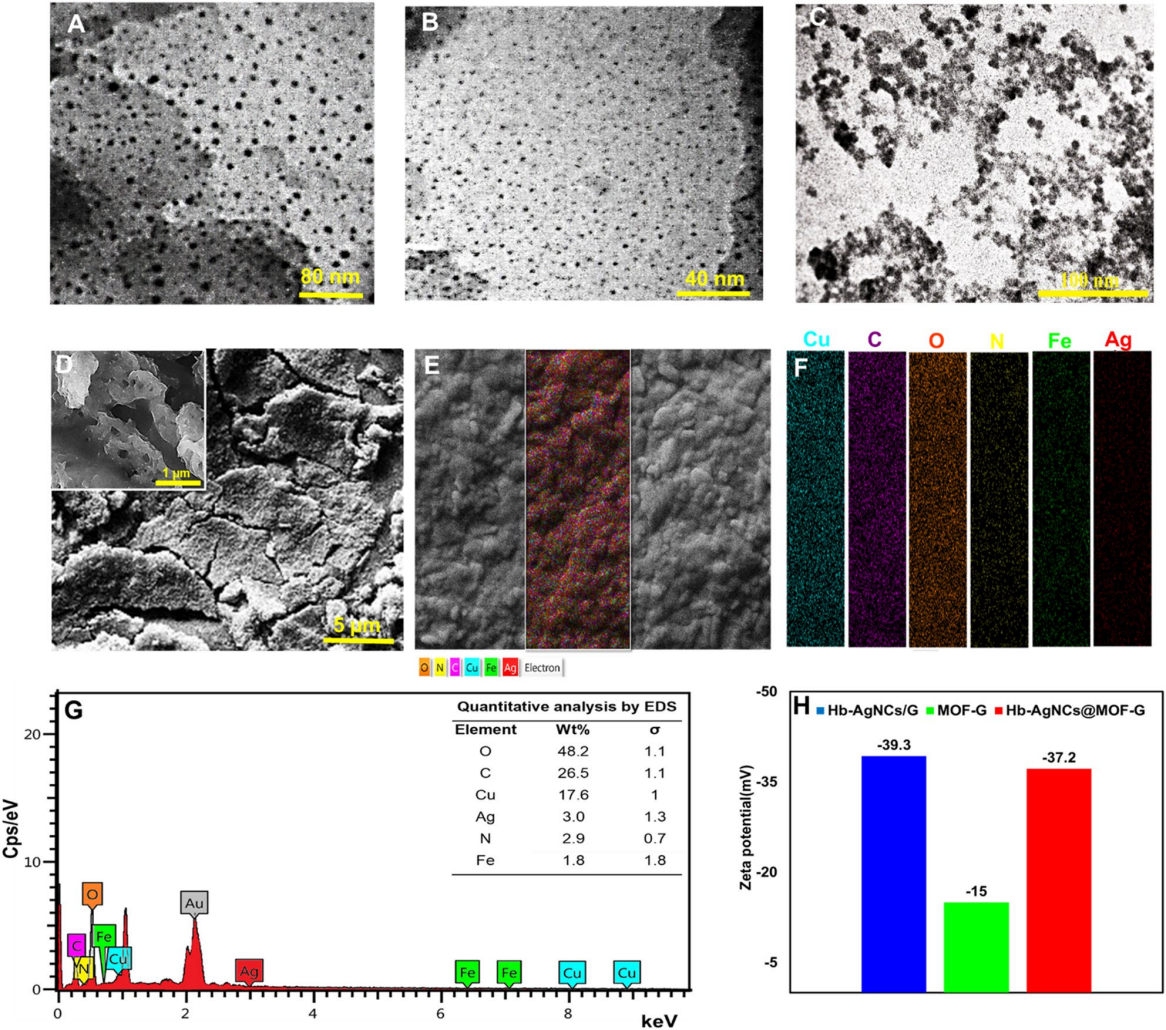
TEM image of (**A**) MOF-G, (**B**) Hb-AgNCs/G and (**C**) Hb-AgNCs@MOF-G. FE-SEM images of (**D**) Hb-AgNCs@MOF-G. (**E** and **F**) EDS mapping and (**G**) elemental analysis images of the nanocomposite. (**H**) Zeta potential measurements of the as-prepared graphene materials.

TEM and FE-SEM images were taken to investigate of the size and morphology of the prepared materials. As seen in Fig. 3A-C, the size of nanohybrid is almost similar to its ingredients, so that the average diameter of Hb-AgNCs@MOF-G, MOF-G and Hb@AgNCs/G were found to be 5.4 ± 0.8 nm, 6.2 ± 1.0 nm, and 4.3 ± 0.7 nm, respectively. Moreover, the TEM results show that Hb-AgNCs@MOF-G consists of MOF-G and Hb@AgNCs crystals decorated into GO exfoliation layers. Notably, in the case of Cu-MOF it can be said that the presence of GO may have particular effects on the production of Cu-MOFs^52^. Really, Cu-MOF "blocks" are linked to GO sheets via interactions between epoxy groups on GO and metal sites of MOF. Furthermore, the epoxy groups prevent MOF aggregation and improve dispersion resulting in the fabrication of well-dispersed and nanosized Cu-MOF^53^.

The results of FE-SEM images illustrated the presence of GO sheets in the developed Hb@AgNCs/MOF-G (Fig. 3D). In addition, considering the porous structure and rough surfaces in SEM images the existence of MOF as well as Hb-AgNCs in the proposed composite could be demonstrated (inset of Fig. 3D)^54^. The presence of pores with large surface area makes antibody molecules more embedded in the composite structure. Figure 3E and F exhibits the elemental mapping of Hb@AgNCs/MOF-G, which has a uniform distribution and is proportional to concentration. Moreover, Fig. 3G depicts the EDS of Hb@AgNCs/MOF-G which confirms the presence of carbon (C), oxygen (O), nitrogen (N), iron (originated from hemoglobin), silver (attributed to AgNCs), and copper (derived from Cu-MOF).

Zeta potential measurements showed that the as-prepared nanocomposite was negatively charged (−37.2 mV) which is near to that of Hb/AgNCs-G (− 39.3 mV) (Fig. 3H) implying high dispersity of the developed nanohybrid. This could be supported by large size of Hb with 574 AA that can stabilize the exfoliated graphene nanosheets and efficiently interacts and hybrids with MOF-G on one hand and effectively cover the AgNCs and inhibit cluster aggregation on the other hand, allowing higher stability and dispersity in aqueous solution^29^. Notably, against the negatively charged both components, their combination (Hb/AgNCs-G and MOF-G) was successful similar to some previously developed composite materials^55–57^.This could be due to different interactions especially hydrogen bonding and π-π stacking effect between Hb protein aromatic amino acids, MOF's aromatic rings and graphene sheets. Moreover, ultrasound through acoustic cavitation has an outstanding ability for the incorporation of organic molecules and metal species into the framework structures and thus instruction of multi-component materials^58^. In this way, energy accumulates in the generated bubbles during ultrasound, and after collapsing, it releases a large energy and so produces micron-droplet leading to easy control chemical reactions. The generated shock waves also cause a significant increase in the mass transfer of materials that is very effective in the synthesis of nanocomposites.

The surface area and porosity were studied using nitrogen adsorption and desorption isotherms for nanocomposite at 77 K. According to the results, pore volume, pore diameter, and the surface area of Hb-AgNCs@MOF-G nanocomposite were obtained 0.11 cm^3^ g^−1^, 1.85 nm, and 1023 m^2^ g^−1^, respectively^59,60^.This is probably due to the effective influence of porous MOF structures as supports for Hb@AgNCs by minimizing the agglomeration of nanoclusters^61^. In fact, the high porosity of MOFs provided a good accessibility for incorporating Hb@AgNCs in the MOF structure. Also, covering the surface of the GO platforms with MOF and Hb@AgNCs leads to an increase in surface area and pore volume. Probably, in this case, in the presence of GO, a dense arrangement of atoms and a porous network is formed. In fact, due to the existence of the porous structure of MOF, the challenge of lack of free space to preserve the molecule is solved^62^.

### Electrochemical performances of label-free immunosensor based on Hb-AgNCs@MOF-G

**Figure 4:**
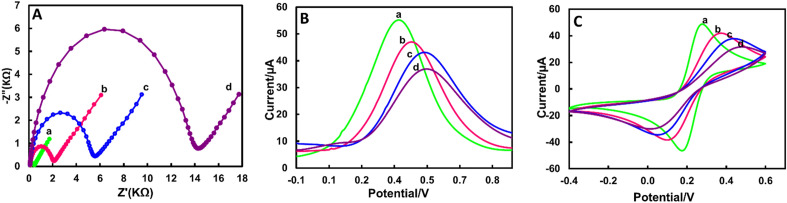
Electrochemical characterization of Nanocomposite by (**A**) Nyquist plots, (**B**) SWV plot and (**C**) CV curves of bare GCE (**a**), GCE/Hb-AgNCs@MOF-G (**b**), GCE/Hb-AgNCs@MOF-G/Herceptin (**c**), GCE/Hb-AgNCs@MOF-G/Herceptin/SKBR3 (**d**), in 0.1 M PBS containing 5 mM K_3_[Fe (CN)_6_]/K_4_[Fe (CN)_6_].

To investigate each step changes of electrochemical biosensor fabrication, EIS technique was used in the presence of the electroactive redox probe [Fe(CN)_6_]^3−/4−^, by which the R_ct_ parameter was measured to control the kinetics of electron transfer. Figure 4A shows the Nyquist plots changes of electrode surface after modification with the developed nanohybrid, Herceptin and HER2 + cancer cells, respectively. The designed nanohybrid was applied as a layer for connect to antibody to identify cancer cells. In Bare GCE, the small semicircle indicates a small R_ct_ value of 0.45 KΩ, suggesting a satisfactory electron transfer process^54^. After the deposition of Hb-AgNCs@MOF-G on GCE, the charge transfer resistance (R_ct_) increases to some extent about 1.65 KΩ, suggesting successful assembly of Hb-AgNCs@MOF-G film on GC electrode surface. In the next step, R_ct_ increased to 5.6 KΩ after covalent cross-linking and immobilization of antibody (Ab) on the modified surface of electrode after activation of carboxyl groups of nanohybrids via carbodiimide/N-hydroxysuccinimide (EDC/NHS) reaction. This is due to the non-electroactive nature of the antibody biomolecule and the prevention of charge transfer. Finally, the particular adsorption between the antigen and the antibody caused the HER2 + cells to be specifically fixed to the electrode during the incubation of the functionalized electrode with cancer cells, which greatly raised the R_ct_ to 14.2 KΩ. Non-electroactive cells block a significant portion of the electrode, preventing electron transport^63^. It represents that the designed Herceptin/Hb-AgNCs@MOF-G/GCE could be used to detect living SKBR3 (Fig. 4A) as a results of particular binding between Ab and the SKBR3 surfaces^64^. Additionally, the construction process of the Hb-AgNCs@MOF-G-based sensor was investigated using Square Wave Voltammetry (SWV) and Cyclic Voltammetry (CV) (Fig. 4B, C). As seen, the peak current responses of SWV and CV detections progressively decreased after each modification step of electrode surface together with a slight peak shift in SWV and an enhanced ∆Ep in CV, which are match satisfactory with EIS results (Fig. 4A).

### Analytical detection of cancer cells based on dual-modal nanohybrid composite structure

**Figure 5:**
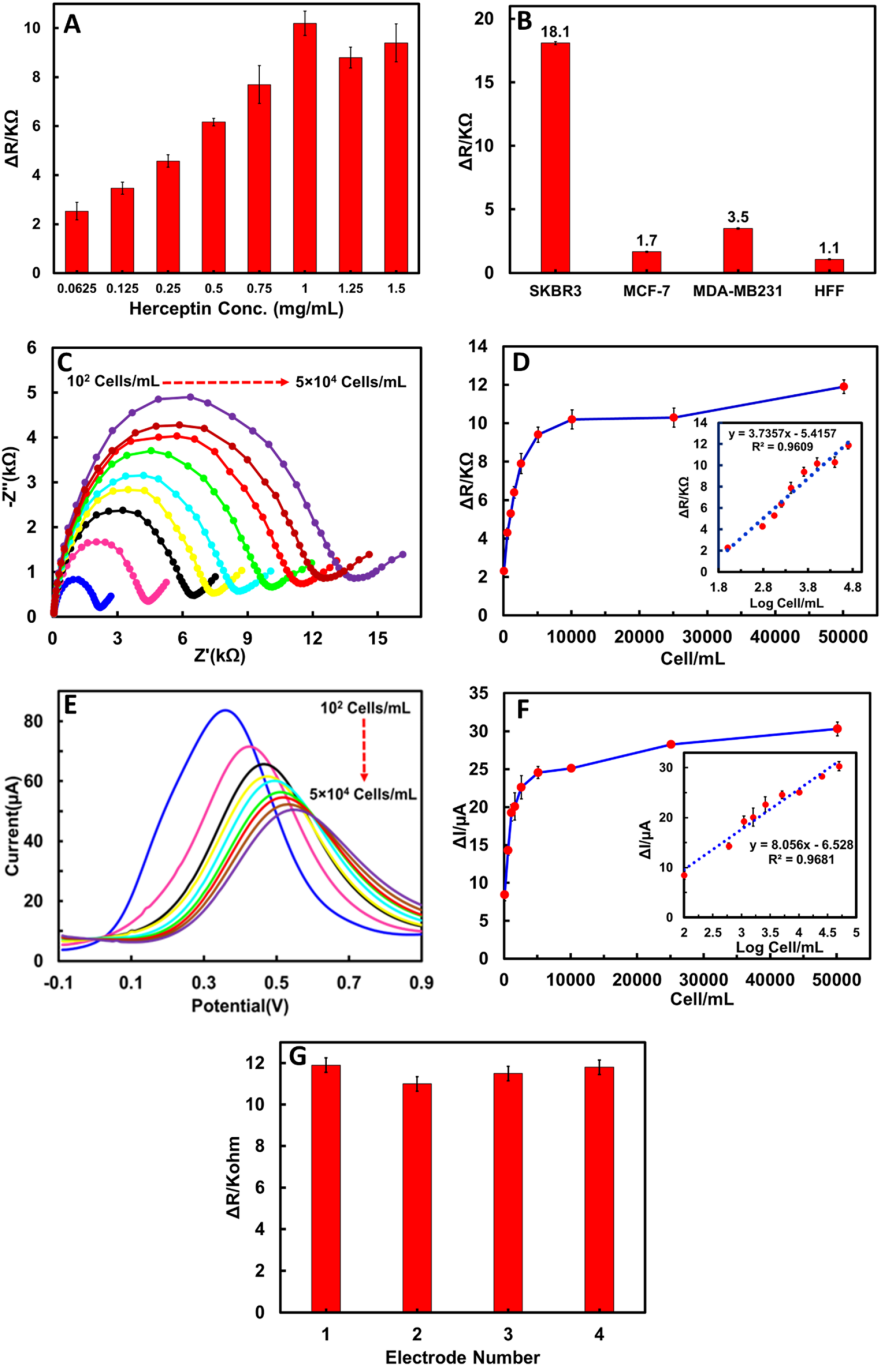
(**A**) Herceptin antibody concentration optimization. (**B**) ΔR_ct_ values of Hb-AgNCs@MOF-G-based electrochemical sensor for SKBR3, MCF-7, MDA-MB231 and HFF cells and concentration of 50 cells, (**C**) EIS responses of the Hb-AgNCs@MOF-G-based sensor with different SKBR3 concentrations (1 × 10^2^, 5 × 10^2^, 1 × 10^3^, 15 × 10^2^, 25 × 10^2^, 5 × 10^3^, 1 × 10^4^, 25 × 10^3^ and 5 × 10^4^ cells/mL). (**D**) Dependence of ΔR_ct_ on the concentration of SKBR3. (**E**) SWV responses of the Hb-AgNCs@MOF-G-based sensor with different SKBR3 concentrations. (**F**) Dependence of ΔI on the concentration of SKBR3. (**G**) Reproducibility of the Hb-AgNCs@MOF-G-based sensor for detecting HER2 + cells with a concentration of 50 cells.

To obtain the optimal condition, the concentration of Herceptin was explored (Fig. 5A). The electrodes were first immobilized with different antibody concentrations and then incubated with 50 SKBR3 BC cells. As depicted, the ΔR_ct_ grew as the antibody concentration increased to 1 mg/mL, indicating the saturation of the Hb-AgNCs@MOF-G/Ab-COOH-modified surface of the GCE electrode by antibody^16^. Therefore, 1 mg/mL was selected as the optimal concentration of Herceptin. Moreover, the response time was evaluated in a specific cell suspension (5 × 10^3^ cells/mL) from 15 to 90 min. As can be seen in Figure S1, ΔR_ct_ increased significantly from 15 to 60 min and then slightly decreased at 90 min; therefore 60 min was selected as the optimal time for next steps.

To study the selectivity of the developed immunosensor for HER2 + cancer cells, various cell lines including HER2-overexpressing SKBR3 breast cancer cells, the HER2-negative breast cancer cells (MCF-7 and MDA-MB231) and normal human fibroblast cells (HFF) were used. For this reason, the modified electrode surface was incubated with a solution containing 50 cells and EIS studies were performed before and after cell incubations (Fig. 5B). The parameter ΔR for SKBR3 cells is 18.1, which is about 5–16 times more than that of other cell lines. In other words, more SKBR3 cells than other cells were linked to the modified electrode surfaces. This is due to the low levels of HER2 expression in MCF-7, MDA-MB231 and HFF cells^65^. Additionally, SKBR3 cells overexpress the HER2 antigen, making them susceptible to the anti-HER2 probe's capture. Therefore, the designed nanocomposite exhibits the strong affinity and selectivity for HER2 + SKBR3 cells (Fig. 5B). In the next step, both EIS as signal on method and SWV as signal off technique were applied to evaluate the efficacy of the fabricated immunosensor upon optimized conditions. For this reason, EIS responses of developed immunosensor were recorded as a function of various concentrations of SKBR3 cells and the limit of detection (LOD) was measured. As exhibited, through an increase in SKBR3 cell concentration, the R_ct_ values increased within the range of 1 × 10^2^–5 × 10^4^ cells/mL (Fig. 5C and D). Inset of Fig. 5D shows the good linearity between the R_ct_ and the logarithm of SKBR3 concentration with a regression equation of ΔR_ct_ (KΩ) = 3.7357x−5.4157 log {SKBR3} (cell/mL) (R^2^ = 0.960) and the LOD of 2 cells/mL. The detecting performance of our proposed nano biosensor toward tumor cells is comparable and even superior to that of most conventional sensors, as demonstrated in Table 1. Similarly, SWV was also used to evaluate the LOD of Hb-AgNCs@MOF-G based sensor toward HER2 + cells to reveal its performance. As seen, with increasing SKBR3 cells, the amount of immunoconjugates increased on the immunosensor, leading to gradually hindering of the redox ferrocyanide probe. Thus, the SWV reduction peak current decreased allowing signal off pathway with the regression equation of ΔI (µA) = 8.056x−6.528 log {SKBR3} (cell/mL) (R^2^ = 0.968) and LOD of 16 cells/mL(Fig. 5E and F). The repeatability of immunosensor was also assessed by four electrodes separately, yielding an RSD of 3.03% (Fig. 5G).

**Table 1:** Different electrochemical biosensors for tumor cell detection.

Analyte	Technique	Nanomaterials	Linear range cells/mL	LOD cells/mL	Reference
MCF-7	EIS	AuNPs	–	10	^66^
SKBR3	stripping voltammetry	Hydrazine–AuNPs–Aptamer Bioconjugate	50–2 × 10^4^	26	^16^
CTCs	I–V response	Dendrimer-Au Nanoparticle	3 × 102–10^3^	80	^67^
CTCs	DPV^a^	PEG-MoS2 NF^s^b@gelatin	50 -10^6^	15	^63^
MDA-MB-231	EIS	HA-BSA-GNPs ^c^	2 × 102–3 × 10^5^	128	^68^
A549	EIS	PDA NPs^d^	1 × 102–1 × 10^5^	25	^69^
CT26	EIS	Cr-MOF@CoPc^e^	50–1 × 10^7^	36	^70^
MCF7	EIS	CD@CuCoPBA^f^	5 × 102–1 × 10^5^	80	^71^
MCF7	EIS	Apt-DTNs^g^	50–1 × 10^6^	5	^72^
MCF-7	LSV^h^	NGQDs/PHA-L^i^	5–1 × 10^6^	1	^73^
MCF7	DPV^a^	CDH Pd − Pt NPs ^k^	150–1.0 × 10^7^	117	^74^
SKBR3	EIS	Hb-AgNCs@MOF-G	1 × 102–5.0 × 10^4^	2	This study
SKBR3	SWV	Hb-AgNCs@MOF-G	1 × 102–5.0 × 10^4^	16	This study

^a^Differential Pulse Voltammetry; ^b^Nanoflakes; ^c^Hyaluronic acid-bovine serum albumin- gold nanoparticles; ^d^Polydopamine nanoparticles; ^e^Cobalt phthalocyanine; ^f^Carbon dots@ CuCo Prussian blue analogue; ^g^Multiaptamer-functionalized DNA tetrahedral nanostructures; ^h^Linear Sweep Voltammetry; ^i^Nitrogen-doped graphene quantum dots/ phytohemagglutinin-L; ^j^Chitosan; ^k^cubic dendritic hollow (CDH) Pd − Pt nanoparticles.

The performance of designed nanocomposite was also separately compared with Hb-AgNCs-G and MOF-G (Figure S2). As seen, the ΔR_ct_ values obtained from nanocomposite are higher than that of components, indicating that the Hb-AgNCs@MOF-G nanocomposite can be an interesting candidate for electrochemical detection of cancer cells.

Considering obtained results and comparing with other reported sensors for detection of cancer cells according to Table 1, it can be concluded that the proposed Hb-AgNCs@MOF/G-based immunosensor will be able to successfully detect HER2 + cancer cells using both signal-on and signal-off methods with the strong analytical performance which could be extended to develop other dual electrochemical biosensing systems. Also, nanocomposite designed in this study had an acceptable performance even compared to noble metal-based nanomaterials like gold metal (Table S1) known as high conductive materials, indicating the conductivity parameter is just not enough for the sensor performance and other parameters such as surface area, surface functional groups, type of target, composite components are important. Moreover, the outcome confirms strong selectivity of the proposed sensor for the precise in vitro diagnosis of BC cells that express the HER2 gene. This could be due to the monoclonal anti-HER2 antibody and its particular antibody interaction with the SKBR3 cell surface that gives the sensor a high level of selectivity. Overall, these findings demonstrate the outstanding stability and high selectivity of the Hb-AgNCs@MOF/G-based nano biosensor for directly detecting SKBR3 cells. It may thus offer a great deal of promise for therapeutic applications.

### Clinical blood sample analysis

To assess the potential clinical application of the proposed biosensor, the standard addition method was applied for one healthy blood and two patient blood specimens. For this, blood samples were spiked by different concentrations of SKBR3 cells and after that incubated directly with the constructed electro interface (Table 2). As shown, the recoveries were acceptable in the range from 79.0 to 113.0% and 79.0 to 111.0% for EIS and SWV methods, respectively. Also, in samples related to breast cancer patients, the sensor was able to detect 2 cells/mL using EIS method, while 10 and 18 cells/mL were detected using SWV method for patient 1 and 2, respectively, indicating the lower sensitivity of SWV method compared to the EIS technique according to the above-mentioned sensitivity obtained for SWV method than EIS method. This shows the detection power of designed sensor in real samples especially based on EIS method. Finally, it can be said that the newly four-component design based on electroactive Hb protein, Ag nanoclusters, Cu-MOF, and graphene can act as the promising nanohybride composite for developing an efficient immuno-biosensor for auxiliary clinical detection of cancer cells in the complicated blood matrix.

**Table 2:** Recovery measurements of SKBR3 spiked in human blood samples for the Hb-AgNCs@MOF/G-based sensor.

	EIS			SWV		
Spiked cell/mL	Found cell/mL	Recovery (%)	RSD (%)	Found cell/mL	Recovery (%)	RSD (%)
Blood sample from healthy donor						
100	106	106.0	9.5	111	111.0	5.1
1000	987	98.7	7.1	1043	104.3	2.3
6000	5688	94.8	7.3	5724	95.4	6.3
16,000	16,864	105.4	2.3	17,003	106.2	12.6
66,000	66,726	101.1	9.9	69,204	104.8	8.3
Blood sample from breast cancer patient 1						
0	2	–	1.4	10	–	2.7
100	81	79.0	5.7	89	79.0	6.1
1000	870	87.0	5.5	809	79.9	10.4
6000	6074	101.2	2.7	6539	108.8	5.4
16,000	18,080	113.0	6.0	16,874	105.4	6.8
Blood sample from breast cancer patient 2						
0	2	–	0.6	18	–	2.6
100	81	79.0	5.1	128	110.0	6.9
1000	1020	101.8	2.7	1055	103.7	6.4
6000	5804	96.7	7.5	5863	97.4	3.0
16,000	17,207	107.5	1.2	16,430	102.5	2.2

## Conclusion

In summary, a new Hb-AgNCs@MOF-G nanohybrid was designed to fabricate a label-free electrochemical immunosensor for rapid and sensitive detection of HER2 + breast cancer cells using the “signal off” SWV and “signal on” EIS strategies. The novelty of this research lies in the successful combination of two distinct materials to create a novel nanomaterial. This newly developed nanomaterial demonstrates excellent activity in electrochemical tests and offers a substantial surface area conducive to biomolecule immobilization. These unique properties highlight its potential for applications in biosensing, particularly in the detection of breast cancer cells. The ability of this biosensor to detect low concentrations of SKBR3 cells, even as low as 2 cells/mL, is one of its most crucial characteristics. This capability is helpful for usage in actual specimens and early diagnosis. The simultaneous presence of both Hb-AgNCs/G and MOF-G creates a synergistic impact that can boost electrochemical performance and enhance antibody-antigen binding endurance that in this case the presence of Hb as an electroactive center and graphene nanosheets as an excellent conductive nanomaterial are very effective. Moreover, the designed Hb-AgNCs@MOF-G nanohybrids offer a large surface area together with highly functional groups for immobilization of anti-HER2, due to lots of aminoacides of Hb protein, their highly ultra-size of nanoclusters, and nanoporous structure of BioMOF leading to an increase of antibody loading on the electrode surface. As a result, the selectivity of the sensor, durability, repeatability, and application are all satisfactory. This approach could be a suitable electrobiosensing model that is easily expandable to label-free rapid diagnosis of other types of tumor cells.

### Supplementary Information

Supplementary Information.

## Data Availability

The datasets used and/or analysed during the current study available from the corresponding author on reasonable request.
