# Supplementary material for: Label-free electrochemical cancer cell detection leveraging hemoglobin-encapsulated silver nanoclusters and Cu-MOF nanohybrids on a graphene-assisted dual-modal probe
Source: Sci Rep. 2023 Dec 11;13:21980. doi: 10.1038/s41598-023-49418-1 (PMC10713537; doi:10.1038/s41598-023-49418-1)
Supplement: Supplementary file 1 — Supplementary Information. [file 41598_2023_49418_MOESM1_ESM.pdf]

Label-Free Electrochemical Cancer Cell Detection Leveraging Hemoglobin-Encapsulated Silver Nanoclusters and Cu-MOF Nanohybrids on a Graphene-Assisted Dual-Modal Probe

Ali-Akbar Zare [a], Hossein Naderi-Manesh [a,b,]*, Seyed Morteza Naghib [c], Mojtaba Shamsipur [d], Fatemeh Molaabasi [e,]*

a Department of Nanobiotechnology, Faculty of Biological Sciences, Tarbiat Modares University, Tehran, Iran

b Department of Biophysics, Faculty of Biological Sciences, Tarbiat Modares University, Tehran, Iran

c Nanotechnology Department, School of Advanced Technologies, Iran University of Science and Technology (IUST), Tehran, Iran

d Department of Chemistry, Razi University, Kermanshah, Iran

e Biomaterials and Tissue Engineering Research Group, Department of Interdisciplinary Technologies, Breast Cancer Research Center, Motamed Cancer Institute, ACECR, Tehran, Iran

***Corresponding Authors:**

Hossein Naderi-Manesh; E.mail: Naderman@modares.ac.ir

Fatemeh Molaabasi; E.mail: molaabasi.fatemeh@yahoo.com

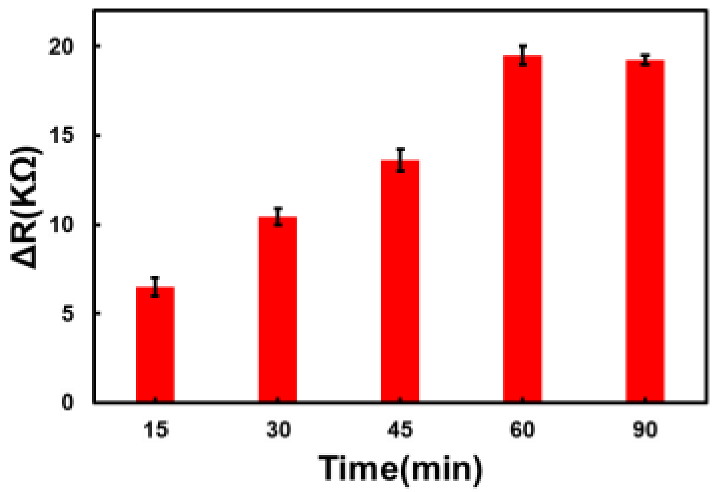

Figure S1: The ΔR_{ct} of 5×10^3 cells/mL on the Herceptin/ Hb-AgNCs@MOF-G /GCE electrode after incubation with SKBR3 cell for different times.

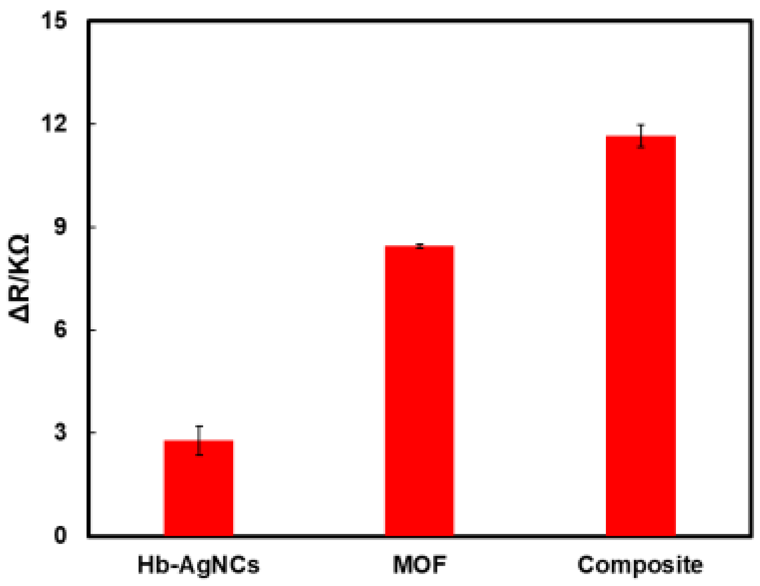

Figure S2: ΔR_{ct} values of Hb-AgNCs-G, MOF-G and Hb-AgNCs@MOF-G -based sensor after incubation with SKBR3 (50 cell).

Table S1: Different electrochemical biosensors based on gold-based nanomaterials for cell detection

Cell	Nanomaterials	Method	Linear range (cells/mL)	LOD (cells/mL)	Reference
A-549 lung cancer	PDAa/AuNPs/rGO	EIS	10^1-10^4	2	1
leukemia K562/B.W cell	AuNPs	DPV	2×10^4 -8×10^5	10^3	2
MCF-7	graphene/AuNPs	DPV	10^2-10^6	6	3
Caco2[c]	AuNPs	chronoamperometric	10^3-5×10^4	4.41×10^3	4
MCF-7	rGO-Cs[b]-AuNP	EIS	10–10^6	4	5
HeLa	PANI-NF/AuNP	EIS	10^4 -6.4×10^6	2×10^3	6

[a] polydopamine; [b] chitosan;[c] Human Colon Adenocarcinoma Cell

1. Linh, N. D., Huyen, N. T. T., Dang, N. H., Piro, B. & Thi Thu, V. Electrochemical interface based on polydopamine and gold nanoparticles/reduced graphene oxide for impedimetric detection of lung cancer cells. *RSC Adv* **13**, (2023).
2. He, F. *et al.* Rapid identification and high sensitive detection of cancer cells on the gold nanoparticle interface by combined contact angle and electrochemical measurements. *Talanta* **77**, (2009).
3. Omidi, M., Yadegari, A., Zali, H., Hashemi, M. & Hasanzadeh, H. Cancer cell detection using electrochemical nanobiosensor based on graphene / gold nanoparticle. *Koomesh* **18**, (2016).
4. Maltez-Da Costa, M. *et al.* Detection of circulating cancer cells using electrocatalytic gold nanoparticles. *Small* **8**, (2012).
5. Shafiei, F., Saberi, R. S. & Mehrgardi, M. A. A label-free electrochemical aptasensor for breast cancer cell detection based on a reduced graphene oxide-chitosan-gold nanoparticle composite. *Bioelectrochemistry* **140**, 107807 (2021).
6. Wang, H. *et al.* Construction of an electrochemical cytosensor based on polyaniline nanofiber/gold nanoparticle interface and application to detection of cancer cells. *Fenxi Huaxue/ Chinese Journal of Analytical Chemistry* **40**, (2012).
